# Conformational Changes in DNA upon Ligand Binding Monitored by Circular Dichroism

**DOI:** 10.3390/ijms13033394

**Published:** 2012-03-12

**Authors:** Yu-Ming Chang, Cammy K.-M. Chen, Ming-Hon Hou

**Affiliations:** 1 Institute of Biological Chemistry, Academia Sinica, Taipei 115, Taiwan; E-Mails: stanley039@yahoo.com.tw (Y.-M.C.); cammychuang@hotmail.com (C.K.-M.C.); 2 Institute of Genomics and Bioinformatics, National Chung Hsing University, Taichung 402, Taiwan

**Keywords:** DNA structure, circular dichroism, conformational changes, DNA-binding drugs, thermodynamic parameters

## Abstract

Circular dichroism (CD) spectroscopy is an optical technique that measures the difference in the absorption of left and right circularly polarized light. This technique has been widely employed in the studies of nucleic acids structures and the use of it to monitor conformational polymorphism of DNA has grown tremendously in the past few decades. DNA may undergo conformational changes to B-form, A-form, Z-form, quadruplexes, triplexes and other structures as a result of the binding process to different compounds. Here we review the recent CD spectroscopic studies of the induction of DNA conformational changes by different ligands, which includes metal derivative complex of aureolic family drugs, actinomycin D, neomycin, cisplatin, and polyamine. It is clear that CD spectroscopy is extremely sensitive and relatively inexpensive, as compared with other techniques. These studies show that CD spectroscopy is a powerful technique to monitor DNA conformational changes resulting from drug binding and also shows its potential to be a drug-screening platform in the future.

## 1. Introduction

Circular dichroism (CD) spectroscopy is an optical technique that measures the difference in the absorption of left and right circularly polarized light [1]. This phenomenon originated from the absorption of optically active chiral molecules. CD spectroscopy is very sensitive to the secondary structure of polypeptides and proteins and thus has been employed in many different fields, such as investigation of the proteins’ secondary structure and spectral studies of DNA [2–5]. Recently, the structures of small organic molecules such as oligo-peptides and oligo-nucleotides have been studied with this technique with 180–300 nm ultraviolet light range [6–9].

Structural information on the oligo-nucleotides at atomic level cannot be provided by CD spectroscopy because the theoretical description is very complex [10]. However, CD spectroscopy offers significant cost and sensitivity advantages when compared with other analysis methods. (i) CD measurement is inexpensive, fast and allows comparative studies of related oligo-nucleotides in various conditions; (ii) We could easily titrate oligo-nucleotides with various agents such as acids, salts, alcohols, drugs and compounds to induce DNA conformational isomerizations; (iii) CD spectroscopy can be used to study oligo-nucleotides as well as longer DNA fragments with a 3000–8000 base pair [11]; (iv) CD can be used to make correlations between X-ray diffraction and infrared spectroscopy because it can analyze not only in solutions but also in films [12]; (v) CD shows extreme sensitivity for low concentrations of DNA (20 μg/mL). In addition, CD shows very high sensitivity (it is possible to detect as little as 25 μg oligonucleotides) which made it a useful method for study low solubility samples.

NMR, X-ray diffraction and other conformational analysis methods have many disadvantages, such as the need for large amounts of oligo-nucleotides, short length of DNA molecules, limited experimental conditions (in solution or crystallization form) and a single discrete structure instead of a mixture of isomerizing conformers. Knowing the advantages and disadvantages of different analysis methods, CD is considered to be one of the easiest and convenient methods designed to study the secondary structure of DNA.

Over the last three decades, enormous advances and efforts have been focused on ligand-DNA interactions. The study of ligand-DNA interactions is critical for a complete understanding of replication and transcription processes, which are attractive targets for the rational design of anticancer and antibiotic drugs. Most chemotherapeutic anticancer drugs currently used come from or are derived from DNA-binding ligands which interact with the DNA duplex by three general binding modes: DNA intercalation, groove binding and covalent binding [13]. DNA interacting drugs exhibit cytotoxic activity to tumor cell through preventing DNA relaxation, blocking gene expression and inhibiting DNA replication. The importance of discovering and designing DNA interacting drug is undisputable. The study of drug-DNA interactions and the mechanisms of drug action is highly important for drug discovery of more efficient and specifically-targeted drugs with fewer side effects [14]. In this review, we summarized the studies of CD spectra, monitoring DNA conformational change upon treatment with several DNA-binding ligands, such as the metal derivative complex of aureolic family drugs, actinomycin D (ActD), cisplatin and polyamines.

## 2. Conformational Polymorphism of DNA and DNA-Drug Complexes Monitored by CD

### 2.1. DNA (B-form, A-form, Z-form and Quadruplexes)

In the most common double helical structure, right-handed B-DNA, the helix makes a turn every 3.4 nm. There are about 10 base pairs per turn and +36° rotation per base pair [15]. The long wavelength CD spectrum of the sequentially heterogeneous DNA is conservative, but the CD spectra of synthetic oligo-nucleotides are depended on the primary sequence. B-form oligo-nucleotides are characterized by a positive long wavelength band at about 260–280 nm and a negative band around 245 nm. However, the position and amplitude of the CD bands show markedly differences in terms of sequence diversity [16–18].

A-DNA is α-helical double helix (right-handed) fairly similar to the B-DNA form, but with a shorter and more compact helical structure. A-DNA occurs only in dehydrated samples and favors low water conditions such as found in aqueous ethanol [19]. However, the slight increase in the number of base pairs per rotation and the smaller rise/turn of A-DNA compared to B-DNA results in a deepening of the major groove and preventing access by proteins. The CD spectrum of poly[d(G)]·poly[d(C)] is similar to that of the true A-form DNA and is characterized by a dominant positive band at 260 nm and a negative band at 210 nm [18,20].

Z-form DNA is a left-handed helix with a zigzag phosphate backbone and shows only little difference in width of major and minor grooves. Formation of this structure is generally unfavorable, although certain conditions can promote it: by raising negative super helical stress or under low salt conditions of the molecule rich in purine-pyrimidine sequences [21]. The CD spectrum of Z-form DNA shows an inversion of the CD spectrum of the B-form, and it contains a negative band at about 290 nm, a positive band around 260 nm, and extremely deep negative band at 205 nm [22,23].

Self-complemented guanine-rich oligonucleotides (GROs) are found in many telomeric repeat sequences, and the G-quadruplex DNA has been constructed from quadruple strands of GROs with a building around tetrads of hydrogen-bonded guanine bases. There are two different types of G-quadruplexes; the spectra of parallel G-quadruplex has a dominant positive band at 260 nm, whereas the spectra of anti-parallel G-quadruplex has a negative band at 260 nm and positive band at 290 nm [24,25]. Moreover, both of them have an additional characteristic positive peak at 210 nm [26].

### 2.2. Chromomycin A_3_(Chro)

**Figure 1:**
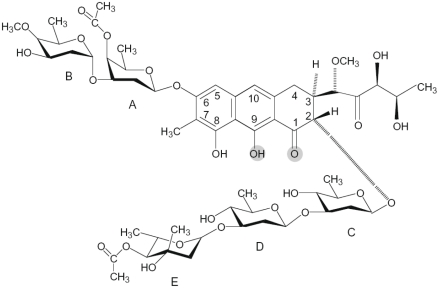
The chemical structure of chromomycin A_3_ (Chro). The oxygen atoms that coordinate the metal ion are shaded.

The anticancer antibiotic chromomycin A_3_ (Chro), a DNA minor groove binding drug which interferes with replication and transcription is a member of the aureolic family of drugs isolated from *Streptomyces griseus* [27–29]. This drug contains di- and trisaccharide moieties connected to a β-ketophenol chromophore via *O*-glycosidic bonds disposed in a 2, 6 relationship about the anthracene ring, with the disaccharide at the two position and the trisaccharide attached at the 6 position (Figure 1). Chromomycin has been used for the treatment of malignant diseases, such as malignancy-associated hypercalcemia and testicular cancer [30].

**Figure 2:**
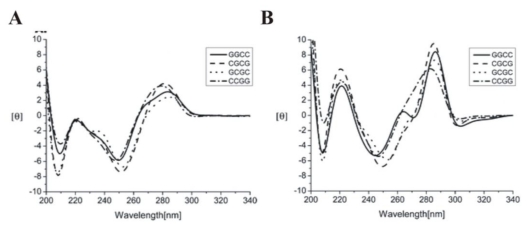
(**A**) CD spectra of hairpin DNA duplexes including GGCC, CGCG, GCGC and CCGG in 25 mM Tris-HCl buffer at pH 7.3 with 50 mM NaCl at 25 °C. The concentration of DNA was 20 μM; (**B**) Normalized sum of the CD spectra of the [(Chro)_2_-Fe^2+^]-hairpin DNA duplex including GGCC, CGCG, GCGC, and CCGG minus [(Chro)_2_-Fe^2+^] dimer in 25 mM Tris-HCl buffer at pH 7.3 with 50 mM NaCl at 25 °C [34]. Reprinted with permission from ref. [34]. (Copyright 2008, American Chemical Society)

Previous optical spectroscopy and structural studies have shown that divalent metal ions, such as Mg^2+^, Fe^2+^, or Co^2+^, are required for Chro to bind to GC-rich oligonucleotide duplexes that are at least three base pairs long [31–33]. The metal ion alternation to influence the effect of aureolic family drugs in disease therapy has been discussed. Previous studies have reported that Chro is capable of forming a dimeric complex via chelation with Fe^2+^ in the absence of DNA. To explore the conformational changes of hairpin DNA duplexes upon binding to the [(Chro)_2_-Fe^2+^] complex, DNA conformations were monitored in the absence or presence of the [(Chro)_2_-Fe^2+^] complex using CD spectroscopy. The DNA tested here form self-complementary hairpin duplexes by using trinucleotides, 5′-TGT-3′, as a loop region. In addition, their stem regions were identical, except for the central portion of four paired nucleotides that have central 2 base-paired (bp) G-tract with directions of either 5′-GC-3′ or 5′-CG-3′ flanked by G and C, *i.e.*, 4 bp G-tract (or A and T, *i.e.*, 2 bp G-tract flanked by A and T). Previously, we have showed that the conformation of [(Chro)_2_-Mg^2+^] remains basically unchanged with and without DNA. Therefore, in the CD spectra of hairpin DNA duplexes complexed to [(Chro)_2_-Fe^2+^], the CD spectra of the [(Chro)_2_-Fe^2+^] complex were all subtracted to minimize the interference from [(Chro)_2_-Fe^2+^]. In Figure 2A, the same profiles were observed for the CD spectra of the hairpin DNA duplexes containing regions of 4 bp G-tract. The CD spectra are similar to typical B-DNA, which showed a band of negative and positive peaks at 250 and 280 nm, respectively [34]. An increase in the CD spectra intensity at ~220 and 280 nm in all hairpin DNA duplexes containing regions of 4 bp G-tract is observed with the presence of [(Chro)_2_-Fe^2+^] complex (Figure 2B). Furthermore, DNA duplex GGCC exhibits the largest change in CD intensity at 280 nm and a smaller peak at 265 nm in the presence of the [(Chro)_2_-Fe^2+^] complex, which are similar to the A-DNA profile.

**Figure 3:**
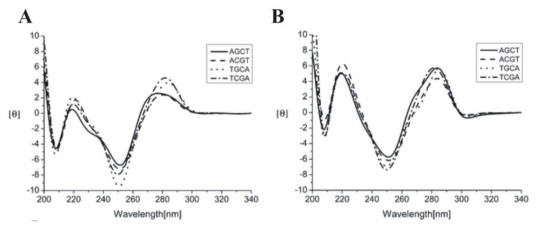
(**A**) CD spectra of hairpin DNA duplexes including AGCT, ACGT, TGCA, and TCGA. The concentration of DNA was 20 μM; (**B**) Normalized sum of the CD spectrum of the [(Chro)_2_-Fe^2+^]-hairpin DNA duplex including AGCT, ACGT, TGCA, and TCGA minus [(Chro)_2_-Fe^2+^] dimer in 25 mM Tris-HCl buffer at pH 7.3 with 50 mM NaCl at 25 °C [34]. Reprinted with permission from ref. [34]. (Copyright 2008, American Chemical Society)

Circular dichroism was also used to measure the interactions between hairpin DNA duplexes that contains regions of 2 bp G-tract flanked by A and T and [(Chro)_2_-Fe^2+^] [34]. Results of CD measurements revealed only slight peak height differences at ~220 and 280 nm between the DNA duplex with/without the treatment of [(Chro)_2_-Fe^2+^] (parts A and B of Figure 3), suggesting subtle structural differences between the two. This is basically consistent with the previous observation that hairpin DNA duplexes containing regions of 2 bp G-tract flanked by A and T exhibited lower equilibrium constants (*K*_a_) upon binding to [(Chro)_2_-Fe^2+^]. In addition, upon binding to [(Chro)_2_-Fe^2+^], the CD spectra of AGCT and ACGT were shifted toward longer wavelengths (~275–280 nm), suggesting a slightly different binding mode.

### 2.3. Mithramycin (Mith)

**Figure 4:**
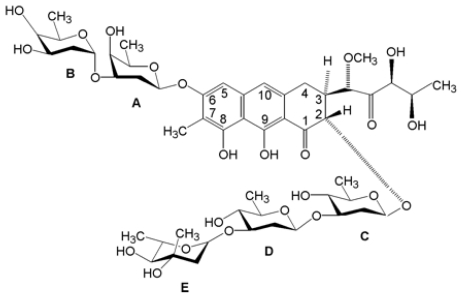
Chemical structure of mithramycin (Mith).

The anticancer antibiotic Mithramycin (Mith) is an RNA synthesis inhibitor and belongs to the aureolic family isolated from *S. griseus* [35]. Mith consists of aglycone ring, a chromomycinone moiety, and sugar residues connected to its either side by *O*-glycosidic bonds (Figure 4). Mith is a DNA-binding antitumor agent that inhibits tumor cell replication and transcription [36,37]. It is currently used in multiple areas of research, several cancer therapies and Paget’s disease [28,38,39]. Recently, Fibach *et al.* [40] revealed that Mith is a potent inducer of fetal hemoglobin production in normal and thalassemic human erythroid precursor cells, and suggested that Mith may be used as a therapeutic agent in certain neoplastic diseases.

**Figure 5:**
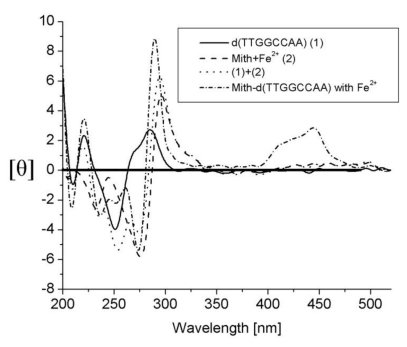
Comparison of the CD spectra of the [(Mith)_2_-Fe^+2^] complex, d(TTGGCCAA)_2_, [(Mith)_2_-Fe^+2^]-d(TTGGCCAA)_2_ complex and the sum of the CD spectra of [(Mith)_2_-Fe^+2^] plus d(TTGGCCAA)_2_ in 20 mM sodium-cacodylate buffer at pH 7.3 with 100 mM NaCl at 25 °C. This figure was adapted from M.-H. Hou *et al.* [41] with permission.

Mithramycin forms a stable dimeric complex by chelating with Fe(II). The structural effects of the [(Mith)_2_-Fe^2+^] complex on the conformation change of d(TTGGCCAA)_2_ DNA duplex has been characterized using CD analyses [41]. As shown in Figure 5, the CD spectra of the [(Mith)_2_-Fe^2+^] complex exhibit negative and positive peak intensities at 275 and 300 nm, whereas the CD spectra of d(TTGGCCAA)_2_ show a similar profile with B-DNA which have a band with negative and positive peaks around 250 and 280 nm. The CD spectra of Mith and DNA signals overlap in the 200–300 nm regions. In addition, the CD spectra of [(Mith)_2_-Fe^2+^]-d(TTGGCCAA)_2_ complex show a higher CD intensity at 287 and 275 nm when compared with the CD spectra sum of the [(Mith)_2_-Fe^2+^] complex plus d(TTGGCCAA)_2_, resulting from a strong exciton-type couplet. It could be used as evidence for the relative binding affinities of the [(Mith)_2_-Fe^2+^] complex toward this DNA oligomer duplex.

### 2.4. Actinomycin D (ActD)

**Figure 6:**
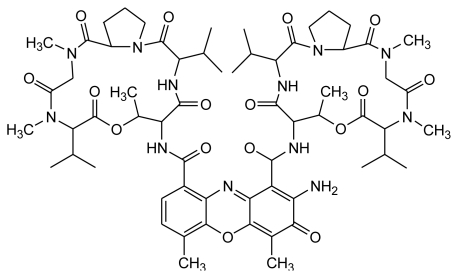
Chemical structure of ActD. The cyclic pentapeptide attached to the quinonoid ring and the benzenoid ring of the phenoxazone group are labeled α and β, respectively.

Actinomycin D (ActD) (Figure 6) is a potent anticancer drug which binds to DNA by intercalating its phenoxazone ring at a GpC step with the two cyclic pentapeptides of the drug located in the DNA minor groove [42,43], thereby interfering with replication and transcription [44,45]. ActD binds specifically to the GpC sequence [46] by forming strong hydrogen bonds between the NH/C=O groups of threonines of ActD and the corresponding N3/N2 sites of adjacent guanine bases of the GpC step. Interestingly, many GpC binding sites are formed when the (CTG)_n_ triplet sequence adopts a hairpin arm (as part of a cruciform) or duplex form, but with alternating T:T mispairs. Studies on the properties of ActD and their interaction with DNA are significantly important in developing new drugs because CTG/CAG triplet repeat expansions (TREs) within genes are associated with various neurological diseases, including Huntington’s disease, myotonic dystrophy, spinocerebellar ataxia and spinal and bulbar muscular atrophy [47–49].

**Figure 7:**
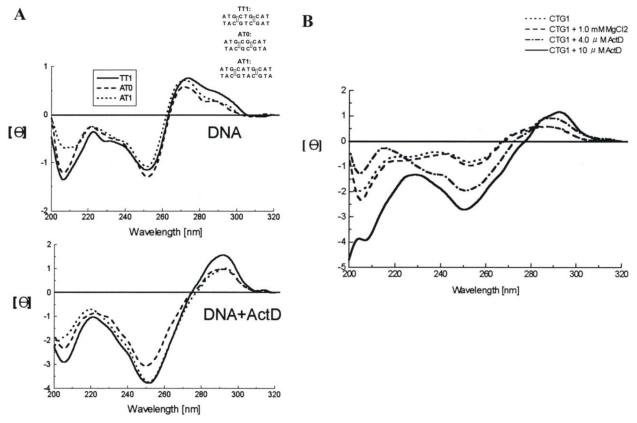
(**A**) CD spectra of AT0, AT1 and TT1 (4 μM) in standard buffer alone (top) and with 10 μM ActD are graphed (bottom). The CD spectra of ActD-DNA complexes were obtained by subtracting the spectrum of ActD, and DNA duplexes used in this study and include AT0, AT1 and TT1 (rectangles represent the binding sites for the phenoxazone ring of ActD); (**B**) CD spectra of CTG1 (1 μM) in the same buffer solution. The CD spectra of ActD-DNA complexes were obtained by subtracting the spectrum of ActD alone. This figure was adapted from M.-H. Hou *et al.* [50] with permission.

Figure 7A shows the CD spectra of DNA duplexes with AT0, AT1 and TT1 in the absence (top) and presence (bottom) of ActD, respectively [50]. The spectral changes observed for the ActD-DNA complexes showed a red shift from 275 to 290 nm, due to a A-type DNA transition upon ActD interaction [51]. In addition, the self-priming sequence CTG1 [5′-(CAG)_4_ (CTG)^16^-3′] can form intra-strand hairpins consisting of both normal and mismatched base pairs, which promote primer-template slippage during DNA replication. The CD spectra of CTG1 showed concomitant increased peak intensity in the presence of 10 μM ActD (Figure 7B). It suggests that conformational changes of long CTG repeats as well as increased stability are induced upon addition of ActD.

### 2.5. Polyamines

Polyamines are ubiquitous polycationic compounds, including spare aliphatic polycationic compounds, and they possess multiple positive charges (two for putrescine, three for spermidine and four for spermine) at physiological pH due to the protonations of their amine groups. Polyamines and the analogues are almost exclusively bound to DNA [52] or RNA [53–55] and are essential regulators of cell growth and gene expression as well as mitosis and meiosis [56]. Information collected recently has shown that polyamines affect protein-DNA interaction [57,58] and modulate the transcription of various genes [59,60]. In some tumor cells, polyamines are regulated poorly, resulting in higher polyamine concentrations than those in normal cells [56]. Furthermore, polyamines are essential materials for catenation of supercoiled DNA by topoisomerase [61,62]. The interaction between polyamines with nucleic acids induces the transition of B-DNA to A-DNA or Z-DNA depending on the conditions [63–65]. In summary, polyamines play important roles in a variety of biological process [56], thus numerous studies on the function of polyamines have been published.

**Figure 8:**
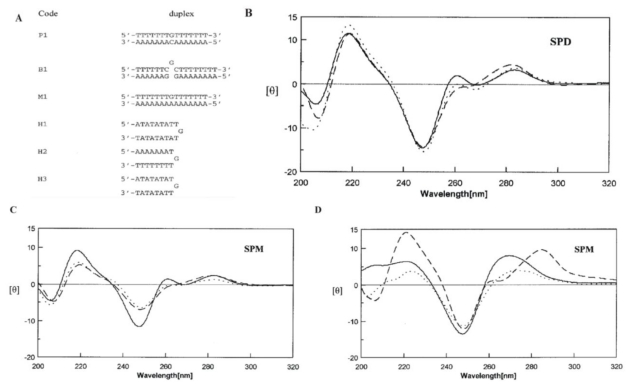
(**A**) DNA duplexes used in the study of polyamines include P1 (a perfect duplex), B1 (a bulged loop duplex), M1 (a mismatched duplex), H1, H2 and H3 (hairpin duplexes); (**B**)(**C**) CD spectra of P1 (solid line), B1 (dotted line) and M1 (broken line) in solution of 20 mM Tris-HCl buffer at pH 7.3 with 5 mM (**B**) spermidine or (**C**) spermine at 10 °C; (**D**) CD spectra of H1 (solid line), H2 (broken line) and H3 (dotted line) in 20 mM Tris-HCl buffer at pH 7.3 with 5 mM spermine at 10 °C. This figure was adapted from M.-H. Hou *et al.* [51] with permission.

Figure 8B and C show the CD spectra of DNA duplexes with normal (P1) and abnormal (M1 and B1) sequences in the presence of spermidine and spermine, respectively. As previous described, DNA duplexes (P1, M1 and B1) have a split peak which includes a large positive peak at 280 nm and a weak positive peak at 260 nm in the region of 260–290 nm of the spectrum similar to the (A)_n_·(T)_n_ sequence [66]. In Figure 8B,C, a band with negative and positive peaks around 245 and 280 nm have been observed for the P1, M1, and B1 CD spectra, which is typical of B-DNA [67] and suggests that DNA duplexes remain as B-DNA conformation. However, addition of spermine causes a decrease in the intensity of both M1 and B1, and to a lesser extent P1 in the region of 240–250 nm.

In Figure 8D, the DNA hairpins H1 and H3 with a (dA·dT)_n_ duplex stem have positive bands at 265 nm in the presence of spermine, which is typical of A-DNA [68]. The observation suggested that an intermediated conformation between B-DNA and A-DNA might be formed for H1 and H3 DNA.

### 2.6. Neomycin

Neomycin belongs to aminoglycoside class of antibiotics that contain two or more aminosugars connected by glycosidic bonds. It has been reported that neomycin could stabilize DNA, RNA, and hybrid triple helices while having little effect on the stabilization of DNA duplex [69]. The competition dialysis studies have shown the binding preference of neomycin toward A-form nucleic acids [70–72]. The selectivity of neomycin most likely originates from its shape complementarity with the A-form nucleic acids and triplex DNA harboring a narrower major groove [71]. In addition, neomycin has been known to bind different RNA structures and is capable of binding tightly to poly(A) [73]. Furthermore, several neomycin conjugates have also been designed and synthesized which performed different nucleic acids selectivity from neomycin. For example, dimeric neomycin-neomycin, neomycin-methidium (NM), and perylene-neomycin conjugates could selectively interact with AT-rich DNA duplex, DNA:RNA hybrid duplex, and human telomeric G-quadruplex, respectively [74–76]. Novel neomycin-Hoechst 33258 conjugate shows remarkable stabilization of DNA duplexes and destabilization of the DNA triplex [77]. BQQ-neomycin and pyrene-neomycin conjugates are more potent in stabilizing DNA helices than neomycin [78,79].

Arya’s group has done several CD studies on these neomycin analogs to monitor drug-DNA interaction. They serially titrated neomycin into a solution of poly(A) and monitored by CD spectroscopy from 300 nm to 200 nm and the result indicated a binding ratio of one neomycin to 10 adenine bases [73]. CD titrations of the oligomer triplex showed an apparent binding site of 2.2 neomycin/triplex or ~6 base triplets/neomycin [80]. The CD titration studies of dimeric neomycin-neomycin conjugate also showed that depending on the structure/sequence of the duplex for AT-rich DNA duplexes, the conjugate binds to DNA with 10–12 base pairs/drug [74]. Moreover, CD experiment results of a series of neomycin conjugates showed that increasing concentration of NM to the DNA:RNA hybrid would result in a gradual increase in the melting temperature [75]. Furthermore, CD spectroscopy titrations of neomycin-Hoechst 33258-Pyrene Conjugate (NHP) showed a significant signal change in the poly(dA)·poly(dT) region, which is represented by a negative band at 248 nm and a positive band at 260 nm [81]. As the molar ratio of NHP:poly(dA)·poly(dT) increased, there is an increasingly positive CD signal in the region of 360 nm [81]. It has also been reported that addition of perylene-neomycin conjugate to the G-quadruplex DNA solution resulted in the changes in the CD intensity of the 295 and 260 nm bands and a slight red shift in the band at 295 nm, which suggests the interaction between the perylene-neomycin conjugate and G-quadruplex DNA [76]. Taken together, CD spectroscopy is an extremely useful tool to monitor specific DNA conformational changes by neomycin.

### 2.7. Cisplatin

Cisplatin is the first member of a class of platinum-containing anti-cancer drugs [82] which is used to treat various type of cancers. Cisplatin forms crosslink interaction with chromosomal DNA to interfere with cell division by mitosis and leads to programmed cell death. This interference activity contributes to Cisplatin’s antitumor activity. Damage to DNA elicits DNA repair mechanisms and activates a programmed cell death process when the damage is too severe to be repaired. Recently, it has been reported that apoptosis would be induced by cisplatin on human colon cancer cells [83].

The guanine N7 position is a favorable site for platinum complexes. Platinum complexes interact with DNA duplex mostly by forming a 1,2-intrastrand cross-link between the N7 atoms of two adjacent guanine bases. According to X-ray crystallographic studies, cisplatin induces the duplex to bend toward the major groove, resulting in significant widening of the minor groove. CD spectroscopy has been used to obtain structural information about the global changes in DNA conformation induced by platinum complexes. It has been shown that the intensity of the positive CD band of B-DNA at 275 nm is increased as a consequence of DNA modification by the complexes containing the *cis*-[PtCl_2_(amine)_2_] unit that reflects distortions in DNA of a nondenaturational nature [84,85]. In addition, the platinum complexes binding to human telomeric G-quadruplex were studied by CD spectroscopy. The non-modified human telomere sequence gives the characteristic CD signature with a maximum at 295 nm and a minimum at 260 nm, and shows an antiparallel “basket” structure [86]. As the pagination level of human telomere sequence increases (up to four platinum atoms per sequence on average), pronounced reduction in the magnitude of the CD is observed along with an increasing shift in the wavelength. In addition, cisplatin caused more pronounced influence on the CD spectra of quadruplexes formed by the model human telomere sequence when compared with transplanting [86].

### 2.8. The Effect of Polyamines on the Metal Derivative Complex of Aureolic Family Drug-DNA Complexes

The paramagnetic metal ions Fe^2+^, Co^2+^, and Cu^2+^ with redo-cycling activities are required for several antibiotics to function properly, such as bleomycin and streptonigrin [37,87]. The positive charge (2+) and radii of these three transition metals are required for Chro-DNA dimeric complexes formation [88]. Spermine exists in the nucleus of cancer cells at millimolar concentrations, and has been reported to interact with transition metals to interfere with the activity of the aureolic family of drugs in cancer cells [89].

**Figure 9:**
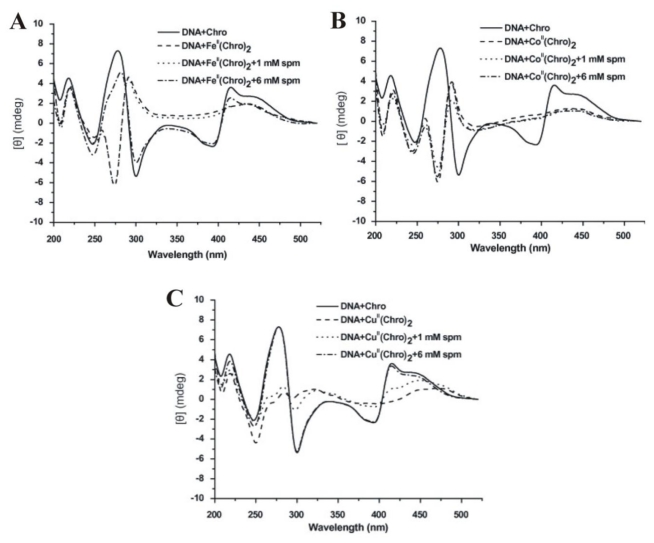
CD spectra of (**A**) [(Chro)_2_-Fe^2+^]-DNA, (**B**) [(Chro)_2_-Co^2+^]-DNA, and (**C**) [(Chro)_2_-Cu^2+^]-DNA in the various concentrations of spermine (0, 1, and 6 mM). The drug concentration was 0.04 mM in a buffer of 20 mM Tris-HCl buffer (pH 7.3). The concentration of DNA was 20 μM. Synthetic hairpin DNA, 5′-TTGGCCAATGTTTGGCCAA-3′, was used in the CD study (hairpin loop underlined). This figure was adapted from W.-J. Lu *et al.* [29] with permission.

**Figure 10:**
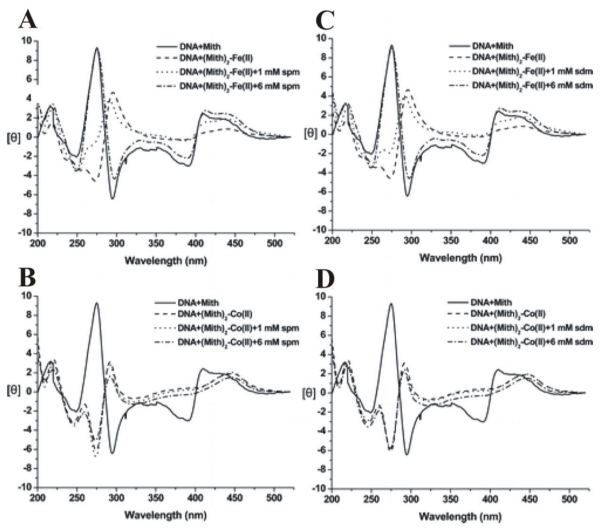
Comparison of the CD spectra of Mith-DNA, (Mith)_2_-metal ion(II)-DNA, and (Mith)_2_-metal ion(II)-DNA in the presence of (**A**,**B**) spermine (spm) and (**C**,**D**) spermidine (sdm) at 1 and 6 mM at 20 °C. Metal ions(II) include Fe^2+^ and Co^2+^. The drug concentration was 0.04 mM in a buffer of 20 mM Tris-HCl (pH 7.3). Reprinted with permission from ref. [90]. (Copyright 2009, American Chemical Society)

To monitor the effect of spermine on the structural integrity of the Fe^2+^-, Co^2+^-, and Cu^2+^-containing dimeric Chro complexes upon DNA binding, the DNA-Chro complexes interaction was examined with increasing concentration of spermine (0, 1, and 6 mM) [29]. As shown in Figure 9, the CD spectra of [(Chro)_2_-Co^2+^] and [(Chro)_2_-Fe^2+^] bound to the DNA duplex exhibited similar spectral features, with negative and positive peaks at 275 and 287 nm, respectively, which are good evidence for an octahedral coordination. However, adding 6 mM spermine to the DNA-[(Chro)_2_-Fe^2+^] complex reverted the CD spectrum to its original profile, suggesting spermine with this concentration is able to inhibit the formation of DNA-[(Chro)_2_-Fe^2+^] complex (Figure 9A). Surprisingly, the same concentration of spermine is unable to interfere with the interaction between DNA and the [(Chro)_2_-Co^2+^] complex (Figure 9B). In the reaction between DNA and [(Chro)_2_-Co^2+^], conformation of DNA changed upon binding with [(Chro)_2_-Co^2+^] and the CD spectra flattened at 275 and 287 nm upon this interaction (Figure 9C). Low concentration of spermine (1 mM) cause a slight decrease at 275 nm in the CD spectrum of the [(Chro)_2_-Cu^2+^]-DNA complex, implying that an interaction occurs between them. When the concentration of spermine increased to 6 mM, the DNA-[(Chro)_2_-Cu^2+^] adduct was completely abolished, as demonstrated by an almost completely inverted CD spectrum (Figure 10C). These data provide strong evidence that spermine exhibits a strong affinity to Fe^2+^ and Cu^2+^ chelated by Chro, while it cannot disrupt the [(Chro)_2_-Co^2+^] complex even at a high concentration.

Moreover, the effect of polyamines on DNA interacting with [(Mith)_2_-Fe^2+^] and [(Mith)_2_-Co^2+^] has also been studied using CD spectroscopy [90]. In the experiment, [(Mith)_2_-Fe^2+^] and [(Mith)_2_-Co^2+^] were incubated with hairpin DNA duplexes, allowing interaction to occur in the presence of increasing concentration of spermine or spermidine (0, 1, and 6 mM). There is one Mith DNA-binding site (GGCC) on the tested DNA, 5′-TTGGCCAATGTTTGGCCAA-3′, which also forms a hairpin duplex using 5′-TGT-3′ as the loop region. In Figure 10, the solid line represents the CD spectrum of DNA and Mith monomer complex (DNA + Mith) and their interaction is not induced with the lack of metal ions. However, Fe^2+^ and Co^2+^ ions caused a dramatic change in CD spectral features at 287 and 275 nm, which proved the ability of metal ions to mediate the interaction between Mith and DNA oligomer duplex. A low concentration of spermine or spermidine (1 mM) caused minor intensity changes of [(Mith)_2_-Fe^2+^]-DNA at 275 and 287 nm, suggesting the complex was partially disrupted. A high concentration of spermine or spermidine (6 mM) was able to compete with Fe^2+^ for the interaction with the complex as the CD spectrum of [(Mith)_2_-Fe^2+^]-DNA was found to completely revert to the same profile as DNA + Mith. On the other hand, the presence of polyamines does not create a different CD spectra of the [(Mith)_2_-Co^2+^]-DNA complexes. Thus, the copper ion in the [(Mith)_2_-Co^2+^]-DNA complex is more resistant to the interaction competition of polyamine.

## 3. Conclusion

The purpose of this short review was to summarize the characteristics of CD spectra of important conformations of DNA and to demonstrate that CD spectroscopy is a useful way to monitor ligand-induced DNA conformational change. The recent development of CD-based technologies has made it possible to directly monitor the binding of ligands and compounds to a variety of DNA (Table 1). In these reports, CD spectroscopy is extremely useful in conformational studies of nucleic acids and for specific ligands that recognize DNA sequences and structures with high selectivity. More reliable studies, related to DNA-binding drugs, may emerge from ongoing experiments using CD spectrum technique.

## Figures and Tables

**Table 1:** CD-based studies of biospecific interaction analysis between DNA-binding ligands and target DNA sequences.

Drugs	Sequence Specificity	Target Sequence
**Anticancer drug**		
Chromomycin A_3_	G/C-rich sequence	d(TTGGCCAA)_2_
	G/C-rich sequence	d(TTGGCCAA)_2_
Mithramycin	G/C-rich sequence	d(TTGGCCAA)_2_
Blenomycin	G/C-rich sequence	d(CCCCAGCTGGGG)
Actinomycin D	CTG triplet repeat sequence	d[(CAG)_4_·(CTG)_16_]
	telomeric G-quadruplex	d[GGG(TTAGGG)_3_]
CC-1065	AT-rich sequence	d(GAATT)
Doxorubicin	telomeric G-quadruplex	d[GGG(TTAGGG)_3_]
Sabarubicin	telomeric G-quadruplex	d[GGG(TTAGGG)_3_]
Streptonigrin	little sequence specificity	d(GCATGC)_2_
*cis*-Platin	GC-rich sequence	poly(dG)·poly(dC)
SN-6999	AT-rich sequence	poly(dA)·poly(dT)
SN-18071	AT-rich sequence	poly(dA)·poly(dT)
Rebeccam ycin	sequences containing GpT (ApC) and TpG (CpA) steps	d(TGTTACGTT)_2_
**Antiviral drug**		
Lamivudine	little sequence specificity	Calf thymus DNA
Valacyclovir	little sequence specificity	Calf thymus DNA
isatin-*β*-thiosemicarbazone	little sequence specificity	Calf thymus DNA
Tartrazine	little sequence specificity	Calf thymus DNA
bis-netropsin	AT-rich sequence	poly(dA)·poly(dT)
Distamycin	AT-rich sequence	poly(dA)·poly(dT)
SN-7167	AT-rich sequence	d(CGCGAATTCGCG) _2_
Berenil	AT-rich sequence	d(G_4_A_4_G_4_-[T_4_]*-C_4_T_4_C_4_-[T_4_]*-G_4_T_4_G_4_)
**Antimicrobial drug**		
Neomycin	A-form nucleic acids	16S A-site rRNA
Neomycin dimer	AT-rich sequence	d[5′-A_12_-x-T_12_-3′]
NHP	AT-rich sequence	poly(dA)·poly(dT)
NM	DNA:RNA hybrids	poly(dA)·poly(rU)
perylene-neomycin conjugate	G-quadruplex DNA	5′-d[AG_3_(T_2_AG_3_)_3_]
Mitomycin C	GC-rich sequence	poly(dG) · poly(dC)
Phenanthrolin	little sequence specificity	Calf thymus DNA
Nogalamycin	little sequence specificity	Calf thymus DNA
Enrofloxacin	little sequence specificity	Calf thymus DNA
Neocarzinostatin	sequence-specific bulged DNA	
**Others**		
Polyamine	Major groove of GC-rich duplexes	poly(dG)·poly(dC)
	Minor groove of AT-rich duplexes	poly(dA)·poly(dT)
Pentamidine	AT-rich sequence	poly(dA)·poly(dT)

* [T_4_] represents a stretch of four thymine residues.
